# Calcineurin Inhibition at the Clinical Phase of Prion Disease Reduces Neurodegeneration, Improves Behavioral Alterations and Increases Animal Survival

**DOI:** 10.1371/journal.ppat.1001138

**Published:** 2010-10-07

**Authors:** Abhisek Mukherjee, Diego Morales-Scheihing, Dennisse Gonzalez-Romero, Kristi Green, Giulio Taglialatela, Claudio Soto

**Affiliations:** 1 Mitchell Center for Alzheimer's Disease and Related Brain Disorders, Department of Neurology, University of Texas Houston Medical School, Houston, Texas, United States of America; 2 Department of Neurology, University of Texas Medical Branch, Galveston, Texas, United States of America; 3 Department of Neuroscience and Cell Biology, University of Texas Medical Branch, Galveston, Texas, United States of America; University of Edinburgh, United Kingdom

## Abstract

Prion diseases are fatal neurodegenerative disorders characterized by a long pre-symptomatic phase followed by rapid and progressive clinical phase. Although rare in humans, the unconventional infectious nature of the disease raises the potential for an epidemic. Unfortunately, no treatment is currently available. The hallmark event in prion diseases is the accumulation of a misfolded and infectious form of the prion protein (PrP^Sc^). Previous reports have shown that PrP^Sc^ induces endoplasmic reticulum stress and changes in calcium homeostasis in the brain of affected individuals. In this study we show that the calcium-dependent phosphatase Calcineurin (CaN) is hyperactivated both *in vitro* and *in vivo* as a result of PrP^Sc^ formation. CaN activation mediates prion-induced neurodegeneration, suggesting that inhibition of this phosphatase could be a target for therapy. To test this hypothesis, prion infected wild type mice were treated intra-peritoneally with the CaN inhibitor FK506 at the clinical phase of the disease. Treated animals exhibited reduced severity of the clinical abnormalities and increased survival time compared to vehicle treated controls. Treatment also led to a significant increase in the brain levels of the CaN downstream targets pCREB and pBAD, which paralleled the decrease of CaN activity. Importantly, we observed a lower degree of neurodegeneration in animals treated with the drug as revealed by a higher number of neurons and a lower quantity of degenerating nerve cells. These changes were not dependent on PrP^Sc^ formation, since the protein accumulated in the brain to the same levels as in the untreated mice. Our findings contribute to an understanding of the mechanism of neurodegeneration in prion diseases and more importantly may provide a novel strategy for therapy that is beneficial at the clinical phase of the disease.

## Author Summary

Prion diseases are a group of infectious neurodegenerative disorders producing a rapid and devastating clinical deterioration. The disease is 100% fatal and affected patients usually die within one year from the appearance of the first clinical alterations. Currently there is no treatment available for these diseases. The main goal of this study was to show that inhibition of the brain phophatase calcineurin is a therapeutic target for prion diseases. We show that calcineurin is hyperactivated as a consequence of endoplasmic reticulum stress produced by accumulation of misfolded prion protein. Treatment of animals showing the symptoms of prion disease with the calcineurin inhibitor FK506 resulted in a substantial decrease in the severity of clinical signs, an increase in animal survival and a reduction in brain degeneration. These findings indicate that calcineurin might play an important role in prion-induced neurodegeneration and inhibition of this phosphatase might represent a promising novel approach for prion disease therapy.

## Introduction

Prion diseases or transmissible spongiform encephalopathies (TSEs) are neurodegenerative disorders affecting humans and animals alike, which are characterized by the presence of PrP^Sc^, an abnormal, protease-resistant isoform of the cellular prion protein, called PrP^C^ [1]. The most common TSE in humans is Creutzfeldt-Jakob disease (CJD) affecting on average one new patient per million people each year [2]. In animals the most common TSE is scrapie, which is an endemic disease affecting sheep and goats for centuries. However, it is the recent appearance of new TSEs that have put prions in the spotlight. These new diseases include variant CJD (vCJD) in humans, bovine spongiform encephalopathy (BSE) in cattle and chronic wasting disease (CWD) in elk and deer. vCJD is the newest and most frightening member of the TSE group. Its appearance in 1996 has been undoubtedly linked to the BSE outbreak and despite that the number of new cases of vCJD seems to be decreasing, there is an enormous concern of secondary transmission of vCJD among humans [3]–[5]. Indeed, recent reports have indicated the propagation of the disease through blood transfusion [6], [7].

At this time there is no effective treatment or cure for TSE [8], [9]. The disease is inevitably fatal and affected people usually die within months of the appearance of the first clinical symptoms. Assuming that the hallmark event in the disease is the conversion of PrP^C^ into PrP^Sc^, a reasonable therapeutic target would be to prevent PrP misfolding and prion replication. This approach has been extensively attempted by many research groups and some compounds have been identified with the capability to decrease prion replication and delay the onset of the clinical disease [1], [9], [10]. However, these compounds produce benefit only when they are administered during the pre-symptomatic stage of the disease, long before the appearance of clinical symptoms. It is likely that compounds interfering with prion replication would have little or no benefit to patients with already established clinical disease, since at the time clinical symptoms appear there is substantial brain damage. It seems that a treatment aimed at patients with established symptoms of CJD would need to attack the cellular pathways implicated in brain damage. This is precisely the major goal of this study, which comes from our recent studies of the mechanism of neurodegeneration in prion diseases.

We have previously shown that purified PrP^Sc^ from scrapie-infected brains is able to induce cell death in primary neuron cultures [11]. Our studies demonstrated that the cellular pathway controlling the induction of apoptosis involves stress of the endoplasmic reticulum (ER) [11]. The first alteration observed when cells were exposed to PrP^Sc^ consisted of the sustained release of calcium from the ER followed by the induction of the unfolded protein response (UPR). The UPR consists of the up-regulation of several molecular chaperones and clearance mechanism to attempt correcting the protein misfolding problem [12]. In support of these *in vitro* observations, histological and biochemical analysis of brains from scrapie-sick mice and from humans affected by sCJD and vCJD demonstrated the presence of activated caspases and the induction of ER-stress inducible chaperones in brain areas exhibiting extensive neuronal death [11], [13], [14]. In the present study we show that an additional consequence of calcium release from the stressed ER is the hyperactivation *in vitro* and *in vivo* of a key protein, termed calcineurin (CaN). CaN is a phosphatase of type 2B highly abundant in the brain that has been implicated in the regulation of synaptic plasticity, memory and neuronal death [15]. CaN immunoreactivity is observed exclusively in neurons in various brain regions, but not in glial cells, including astrocytes, oligodendrocytes, microglia and ependymal cells both in humans and rodents [16], [17]. Subcellular localization studies by electron microscopy showed that calcineurin was found in dendrites including postsynaptic densities, cell bodies, spines, axons and terminals [16]. The activity of this enzyme is regulated by the Ca^2+^-calmodulin complex. Optimum activity of CaN is required to maintain the proper phosphorylation state of different important targets, like apoptosis inducer BAD or transcription factor CREB. Hyper-activation of CaN reduces the phosphorylation of BAD [18], which then disassociate from the scaffolding protein 14-3-3 and interacts with Bcl-Lx or other Bcl2 family proteins located in the mitochondrial membrane. As a result cytochrome C is released in the cytoplasm, leading to caspase activation and finally causing apoptosis. On the other hand dephosphorylated by hyper-activated CaN, CREB is not able to translocate into the nucleus to act as a transcription factor to regulate expression of different genes required for synaptic plasticity [19].

To study the potential role of CaN in prion-induced neurodegeneration and to assess the possibility of inhibiting this phosphatase as a putative target for therapeutic intervention, we treated prion infected animals with the FDA-approved CaN inhibitor FK506. FK506 (also known as Tacrolimus and Prograf) is a natural product produced by the fungus Streptomyces tsukubaensis, marketed to prevent transplant rejection [20]. By inhibiting CaN, the drug suppresses both humoral and cellular immune responses [21]. Our results show that mice receiving FK506 after the onset of the clinical phase of prion disease exhibited improved motor activity and coordination, lived longer and had a decreased level of brain degeneration when compared to untreated prion infected animals. These findings suggest that inhibition of CaN activity in the brain may be a promising new approach for the treatment of prion diseases.

## Results

### PrP^Sc^-induced neurodegeneration is mediated by CaN activation *in vitro* and *in vivo*

**Figure 1:**
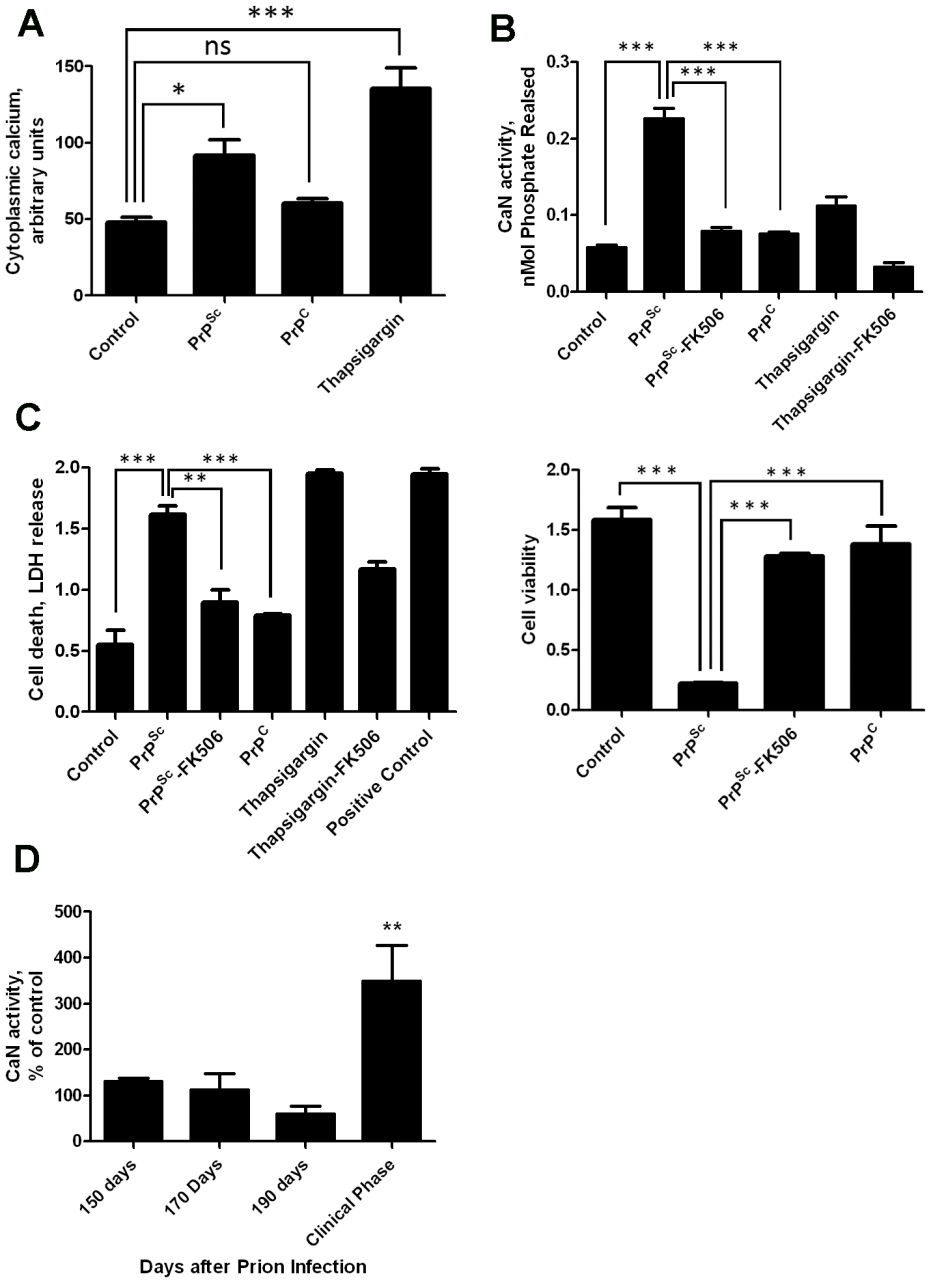
PrP^Sc^ neurotoxicity is mediated by CaN activation. Panels A, B and C show the results of *in vitro* experiments in which 200 nM of purified PrP^Sc^ was added to the medium of 1×10^5^ N2A neuroblastoma cells. **A**, Calcium concentration in cytoplasm was measured after 20 minutes of exposure to PrP^Sc^ using the Fluo-4 Direct Calcium Assay kit in 96 well plate according to manufacturer's protocol. **B**, Cells were treated for 36 h with 200nM PrP^Sc^ in the presence or the absence of 10µM FK506. As controls, cells were treated with 200 nM of purified recombinant PrP^C^ or with 20µM Thapsigargin (with or without 10 µM FK506). CaN activity was measured using the Calcineurin Cellular Activity Assay kit from Calbiochem according to manufacturer's protocol. **C**, The effect of PrP^Sc^ on cell death (left panel) or cell viability (right panel) was measured after 48 hours of incubation (except for the experiment with Thapsigargin that was measured 24 hours after addition of the chemicals). The LDH and MTT assay were performed using the LDH assay kit (Promega) and Cell proliferation assay kit (Roche), respectively, according to the manufacturers' protocol. In case of the cell death assay the positive control, which represents the maximum amount of LDH release, was done by addition of a final concentration of 2% tween20 to the culture medium. All experiments in panels A, C and C were done in triplicate and the values correspond to the average ± standard error. In these experiments the data was analyzed by one-way ANOVA, followed by the Tukey's Multiple Comparison post-test to estimate the significance of the differences. **D**, To study the potential role of CaN in prion diseases we measured the level of CaN activity in animals at different stages of prion disease. For this purpose, groups of 3 mice were sacrificed at different times after i.p. inoculation with RML prions. The brain was collected, homogenized and the CaN activity measured as described in Methods. CaN activity is expressed as a percentage of the value obtained in non-infected wild type mice. There were not differences in CaN activity in the brain of control (non-infected) animals during the age range studied (150–250 days). The values used for normalization corresponded to an average of all the animals tested. The differences were analyzed by unpaired student t-test. * P<0.05; ** P<0.01; *** P<0.001.

Our previous results showed that PrP^Sc^ accumulation induced ER stress [11]. One of the alterations produced by ER stress is the change of calcium homeostasis. Indeed, exposure of N2A mouse neuroblastoma cells to brain-isolated PrP^Sc^ results in the increase of cytoplasmatic calcium (Fig. 1A), which as shown before is coming from the ER [11]. It is well-established that CaN activity is modulated by cytoplasmic calcium concentration. To assess whether neuroblastoma cells exposed to PrP^Sc^ indeed have a higher level of CaN activity, we measured the phosphatase activity in N2A cells treated with 200 nM of PrP^Sc^. The results show that CaN activity is significantly elevated in cells exposed to PrP^Sc^, but not to the same concentration of the natively folded recombinant prion protein (PrP^C^) (Fig. 1B). As positive control we used Thapsigargin, an inhibitor of the sarco/endoplasmic reticulum Ca^2+^ ATPase that increases cytoplasmatic calcium concentration [22]. FK506 (also known as Tacrolimus and Prograf) is a FDA approved drug able to inhibit CaN activity [21]. The PrP^Sc^-induced elevation of CaN activity was efficiently blocked by concomitant addition of FK506 (Fig. 1B).

To assess the effect of PrP^Sc^ and subsequent CaN activation in cell damage, we measured cell death by release of lactate dehydrogenase (LDH) and cell viability by the MTT (3-(4,5-Dimethylthiazol-2-yl)-2,5-diphenyltetrazolium bromide) assay in N2A cells exposed to 200 nM of PrP^Sc^ in the presence or absence of FK506 (Fig. 1C). Cell death produced by treatment with PrP^Sc^ or thapsigargin was significantly decreased by addition of the CaN inhibitor FK506 (Fig 1C, left panel). Indeed, the rate of neuroblastoma cell death after treatment with PrP^Sc^ and FK506 was not significantly different from the control untreated cells, indicating that FK506 completely protected cells from PrP^Sc^ toxic activity. A similar protective effect of FK506 against PrP^Sc^ neurotoxicity was found when cell viability was measured by MTT reduction (Fig. 1C, right panel). Treatment of cells with FK506 in the absence of PrP^Sc^ did not change cell death or viability (data not shown).

To study whether the increase in CaN activity observed *in vitro* as a consequence of PrP^Sc^ formation also occurs under *in vivo* conditions, we measured CaN activity in brain of animals infected with prions. Groups of mice intra-peritoneally infected with the RML prion strain were sacrificed at various time points until the onset of the clinical signs, which under these conditions occurred between 210 and 230 days post-inoculation. A basal CaN activity in brain was detected at all time points during the pre-symptomatic phase, which was not different from the activity found in non-inoculated animals (Fig. 1D). However, at the beginning of the clinical phase of the disease, a ∼3-fold higher CaN activity was found in brain, indicating that the activity of this phosphatase is significantly elevated at the time brain damage and clinical disease occur.

### Inhibition of CaN activity decreases disease severity and increases animal survival

To study whether CaN activity is implicated in the progression of prion disease and whether the inhibition of this phosphatase could be a good target for therapy, groups of mice were treated with FK506 during the clinical phase of the disease. Clinical onset of the disease was carefully measured every other week by two different researchers monitoring the appearance of hunch and trail rigidity, which is the first alteration clearly associated to the disease. When the clinical symptoms were unambiguously observed during 3 consecutive days, infected mice were injected i.p. daily with 5 mg/kg of FK506 (dissolved in saline, containing 1.25% PEG40 Castor oil and 2% ethanol) (n = 14) or with vehicle (n = 14). This dose was chosen based on a toxicology study showing that administration of FK506 at concentrations higher than 10 mg/Kg produced side-effects detectable by behavioral tests (data not shown). As a control we used another immunosuppressant drug, Rapamycin (n = 8), which does not interfere with CaN activity. Treatments lasted until animals died or were sacrificed for analysis.

**Figure 2:**
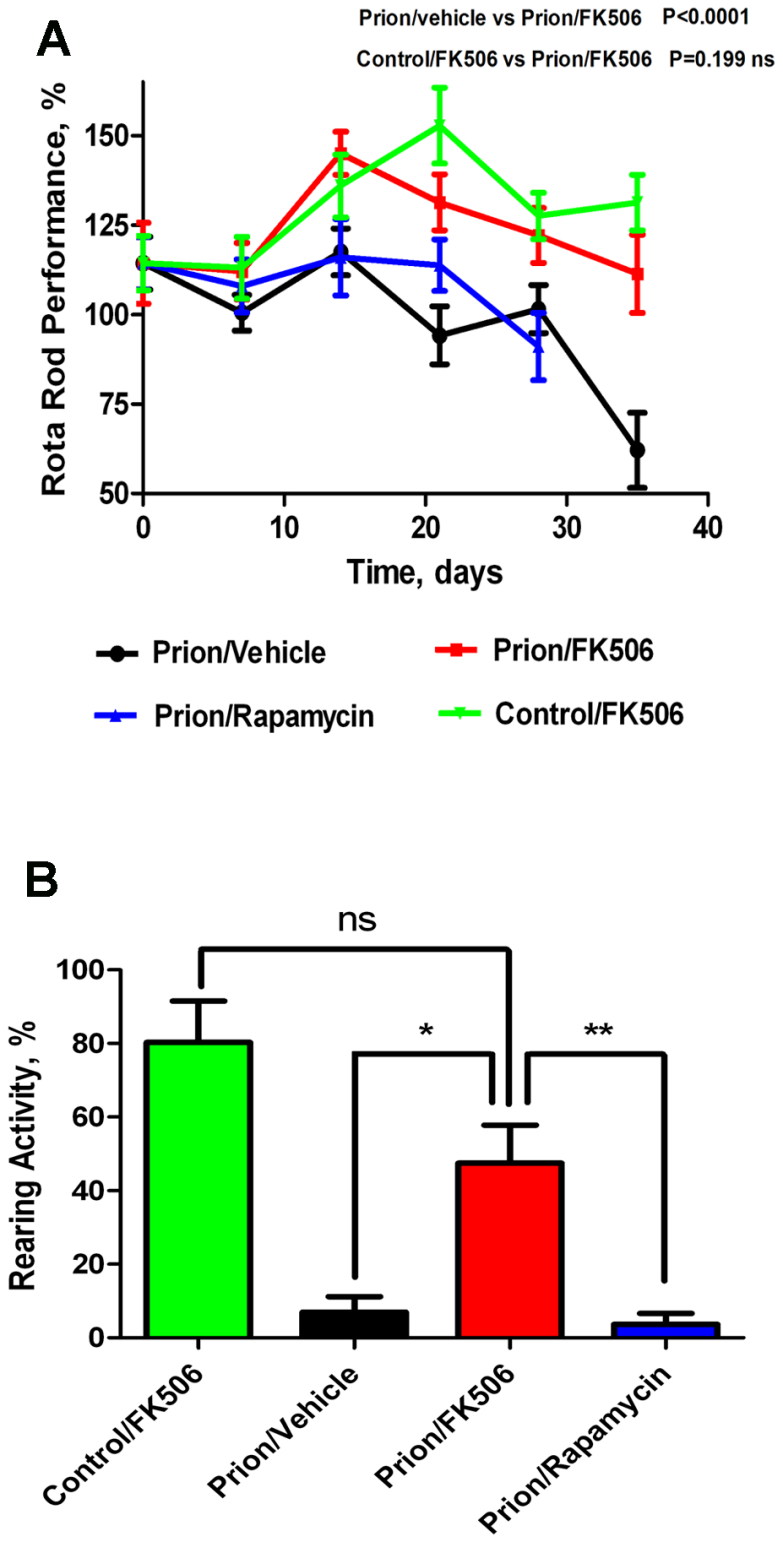
Inhibition of CaN decreases severity of disease clinical alterations. Animals were treated daily with 5 mg/Kg of FK506, rapamycin or vehicle (the buffer used to dissolve FK506) starting at the time they exhibited the first signs of prion disease. Behavioral and motor alterations were measured over time by Rota-rod (**A**) or open field (**B**) tests and compared with age matched wild type animals treated with FK506. Activity was assessed once a week and the initial performance (t = 0) was normalized with respect to the age matched wild type non-infected animals. The locomotor activity in the rota-rod test was measured by placing mice on the rotating rod with an accelerated speed and the total time spent without falling was recorded. The rearing activity in the open field box was measured as the number of times the animal spent in vertical activity during 20s intervals. The graph in panel B shows the results obtained at 21 days after beginning of the treatment. Each value corresponds to the average and standard error (n = 4 for rapamycin and n = 10 for other groups).

Progressive decline in motor coordination and activity is a well documented clinical feature of prion disease [23], which likely is the result of loss of synaptic integrity. Therefore motor alterations during the clinical phase of the disease in animals treated with FK506, rapamycin or vehicle were assessed by using rota-rod (Fig 2A) and open field tests (Fig 2B). The motor coordination in the rota-rod test was measured once a week in a 3 min time interval and was expressed as a percentage of the activity at day 0 (day in which treatment was started). The results showed that prion infected animals treated with vehicle or rapamycin experienced a progressive and significant decline on motor coordination, whereas animals treated with FK506 showed no significant decrease on performance over de 5 weeks period (Fig. 2A). Indeed, the rota-rod performance of the FK506 treatment group was similar to that of non-infected animals (either treated or untreated with FK506), used as control. The slight increase on performance over the time period in control animals probably reflects that animals become more familiarized with the test. The locomotor ability in open field test was measured at day 21 after starting the treatment, by the rearing activity during 20s and corresponds to the time animals spent only on the hind limb. As shown in figure 2B, the rearing activity in prion infected animals was dramatically reduced compared to control non-inoculated animals. Treatment with FK506, but not rapamycin, prevented significantly the locomotor deficiencies (Fig. 2B).

**Figure 3:**
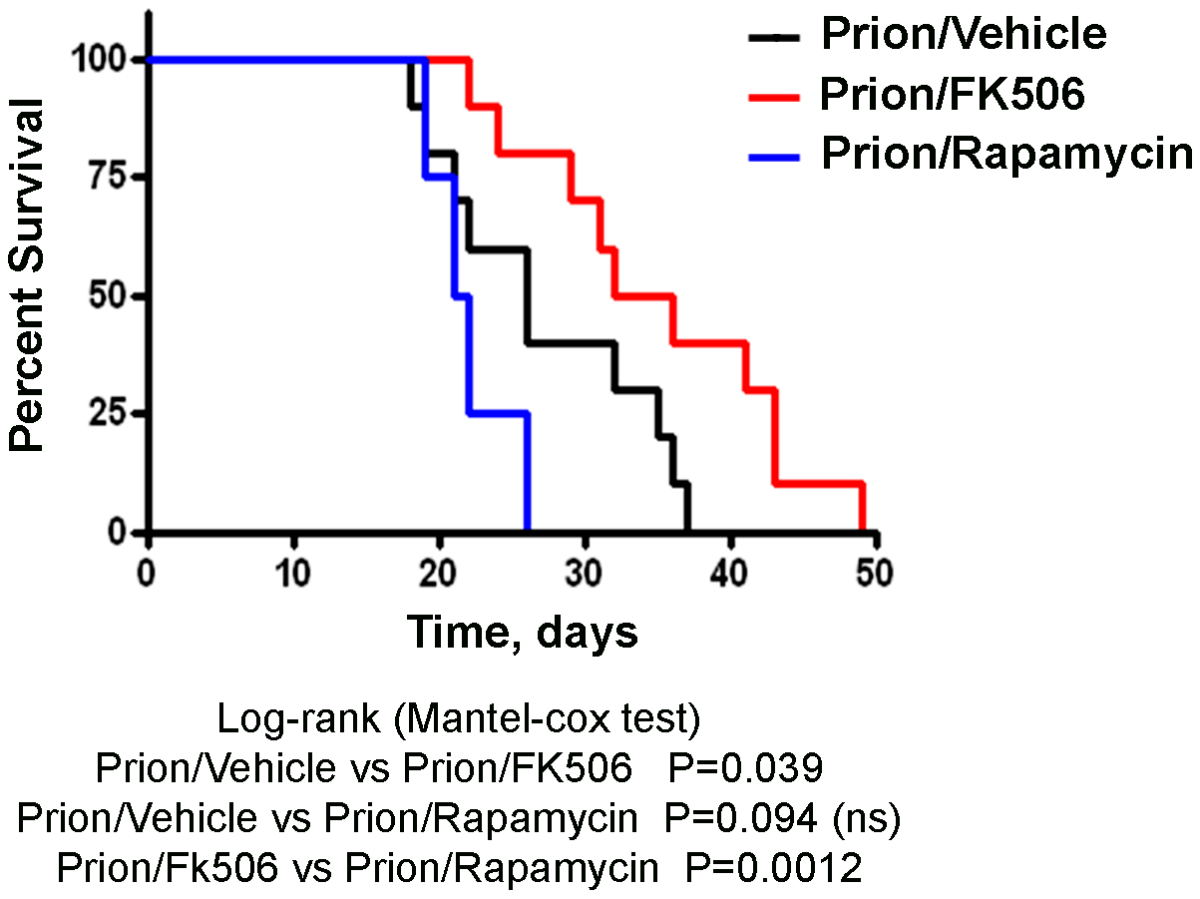
Increase in survival of prion infected animals by treatment with CaN inhibitor. Animals were treated daily with 5 mg/Kg of FK506, rapamycin or vehicle (the buffer used to dissolve FK506 and rapamycin) starting at the time they exhibited the first signs of prion disease. Groups of animals were left to die to determine survival time in each condition. FK506 treated mice (n = 10, red line; average symptomatic phase: 35±2.8 days), treated with rapamycin (n = 4, blue line; average symptomatic phase: 20.9±0.9 days), vehicle group (n = 10, black line; average symptomatic phase: 26.2±2.2 days). Differences were analyzed by the Log-rank (Matel-cox) test.

Next we investigated the survival time of the animals under treatment. Infected mice were left to die naturally and time of death was recorded. Figure 3 shows the survival curve of the group treated with FK506 (n = 10, red line), treated with rapamycin (n = 4, blue line) or the vehicle group (n = 10, black line). The results showed a 35% increase in survival of the group treated with CaN inhibitor compared to the vehicle treated control (P = 0.039, as estimated by the Log-rank Mantel-cox test).

### FK506 treatment does not alter PrP^Sc^ accumulation, but increases pCREB and pBAD

**Figure 4:**
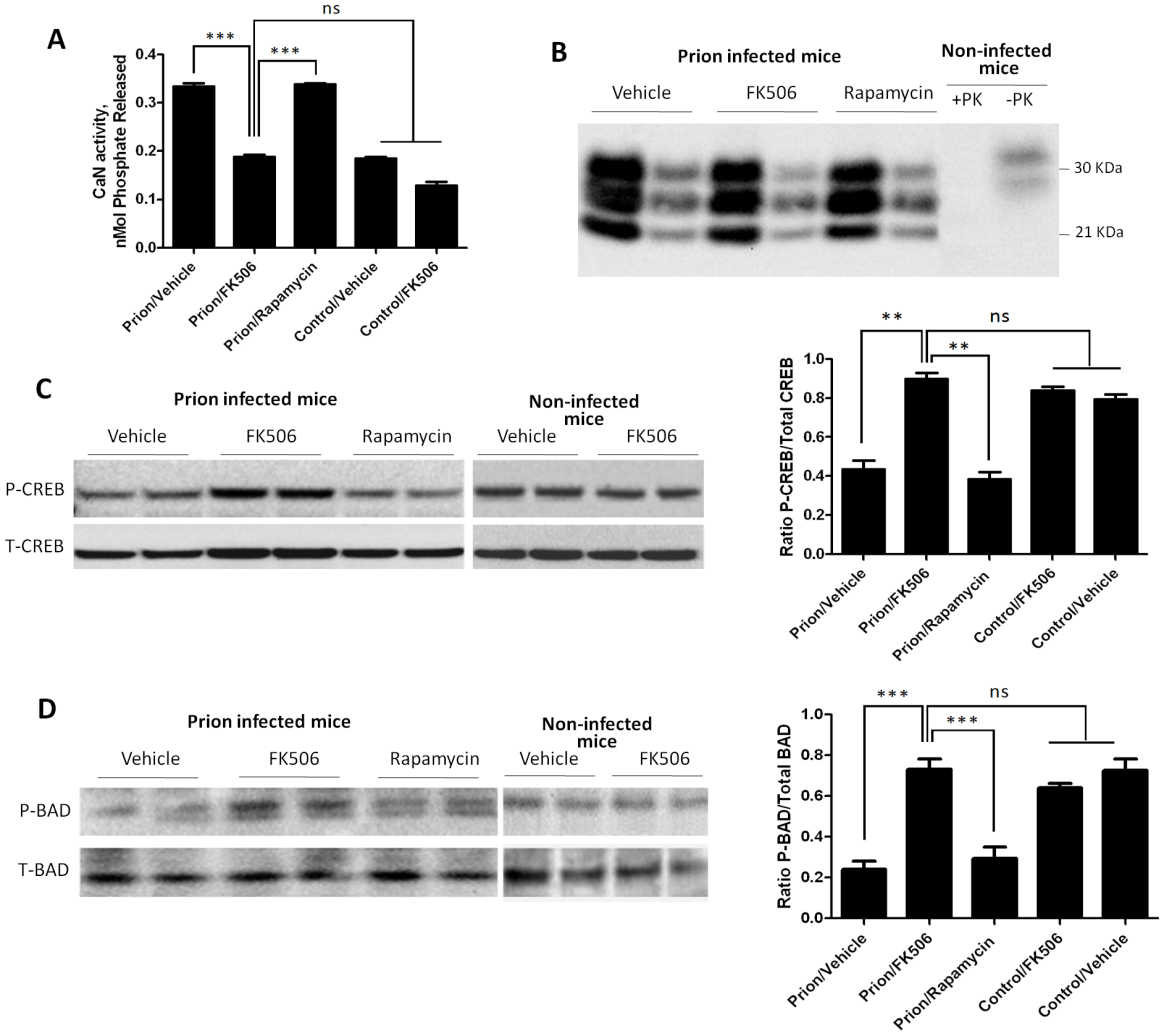
Effect of FK506 treatment on PrP^Sc^ formation, CaN activity and downstream targets. To study the effect of the treatment on neurodegeneration, 4 random animals from each treatment group were sacrificed when mice injected with vehicle reached the stage 5 of the clinical disease. Their brain was collected and one hemisphere was kept frozen for biochemical studies. As controls we used age-matched non-infected animals that received either vehicle or FK506. **A**, levels of CaN activity were measured as described in Methods. The values represent the average ± standard error of 3 determinations. **B**, PrP^Sc^ accumulation was determined by western blot after PK digestion as described in Methods. For each condition, the western blot of a representative animal is shown in two dilutions (5 and 25 fold dilution of 10% brain homogenate). Normal mouse brain homogenate treated in the same way with or without PK digestion was also loaded. **C**, The brain levels of pCREB and total CREB were determined by western blots in animals infected with prions and treated with vehicle, FK506 or rapamycin, as well as in non-infected age-matched mice treated with vehicle or with FK506. The blot shows the results of 2 different animals per group and the graph in the right side display the densitometric analysis of the 4 animals studied in each condition. The values correspond to the average ± standard error. **D**, The levels of pBAD and total BAD were estimated by Western blot and the data densitometrically analyzed. As before, the values in the graph correspond to the average ± standard error of 4 animals per group. For the western blot analysis, the same total protein concentration was loaded in each lane and equal loading was additionally confirmed by developing the membrane with anti-actin antibody. The data was statistically analyzed by One-way ANOVA followed by the Tukey's Multiple comparison post-test. * P<0.05; ** P<0.01; *** P<0.001.

To study the effect of FK506 treatment on neurodegeneration, 4 random animals from each treatment group were sacrificed at the time in which the first mice injected with vehicle reached the stage 5 of the clinical disease (around 20 days after the beginning of the treatment), their brain collected and one hemisphere was kept frozen and the other fixed for histological analysis (see below). Frozen brains were homogenized in TBS with protease inhibitors. The level of CaN activity in total brain was measured as described in Methods. Prion inoculated mice had substantially higher CaN activity in brain than non-inoculated mice (Fig. 4A), supporting the result shown in Fig 1D. The levels of CaN activity in prion inoculated mice were normalized upon treatment with FK506, indicating that the drug is acting as expected in the brain and that the dose used was appropriate.

To measure PrP^Sc^ accumulation in the different group of animals, samples were treated with PK (50 µg/ml for 1h at 37°C) and 1/5 and 1/25 dilutions from the 10% brain homogenate were evaluated by western blot. The result shows that FK506 treatment did not alter PrP^Sc^ accumulation (Fig. 4B), a result consistent with our hypothesis that CaN activation is a downstream event of prion replication. To study the influence of CaN inhibition in the phosphorylation stage of some of the key substrates of the phosphatase involved in controlling synaptic plasticity and neuronal apoptosis, we measured the brain levels of pCREB, pBAD as well as total CREB and BAD. The western blot in figure 4C shows the results for CREB of two representative animals per group and the graph in the right panel represents the quantification of the ratio pCREB/total CREB. The results indicate that scrapie-affected mice receiving vehicle have a 2-fold reduction of pCREB (normalized to total CREB) compared to non-infected animals. Strikingly, treatment with FK506, but not rapamycin, restored the concentration of this phosphorylated transcription factor to reach levels indistinguishable from healthy controls (Fig. 4C). A similar result was observed when the pro-apoptotic BAD protein was analyzed. Indeed, prion affected mice have a 3–4 fold reduction of pBAD/total BAD ratio compared to controls. Again, the phosphorylation state of this important protein was restored upon treatment with FK506 (Fig. 4D).

### CaN inhibition reduces neurodegeneration in prion affected mice

**Figure 5:**
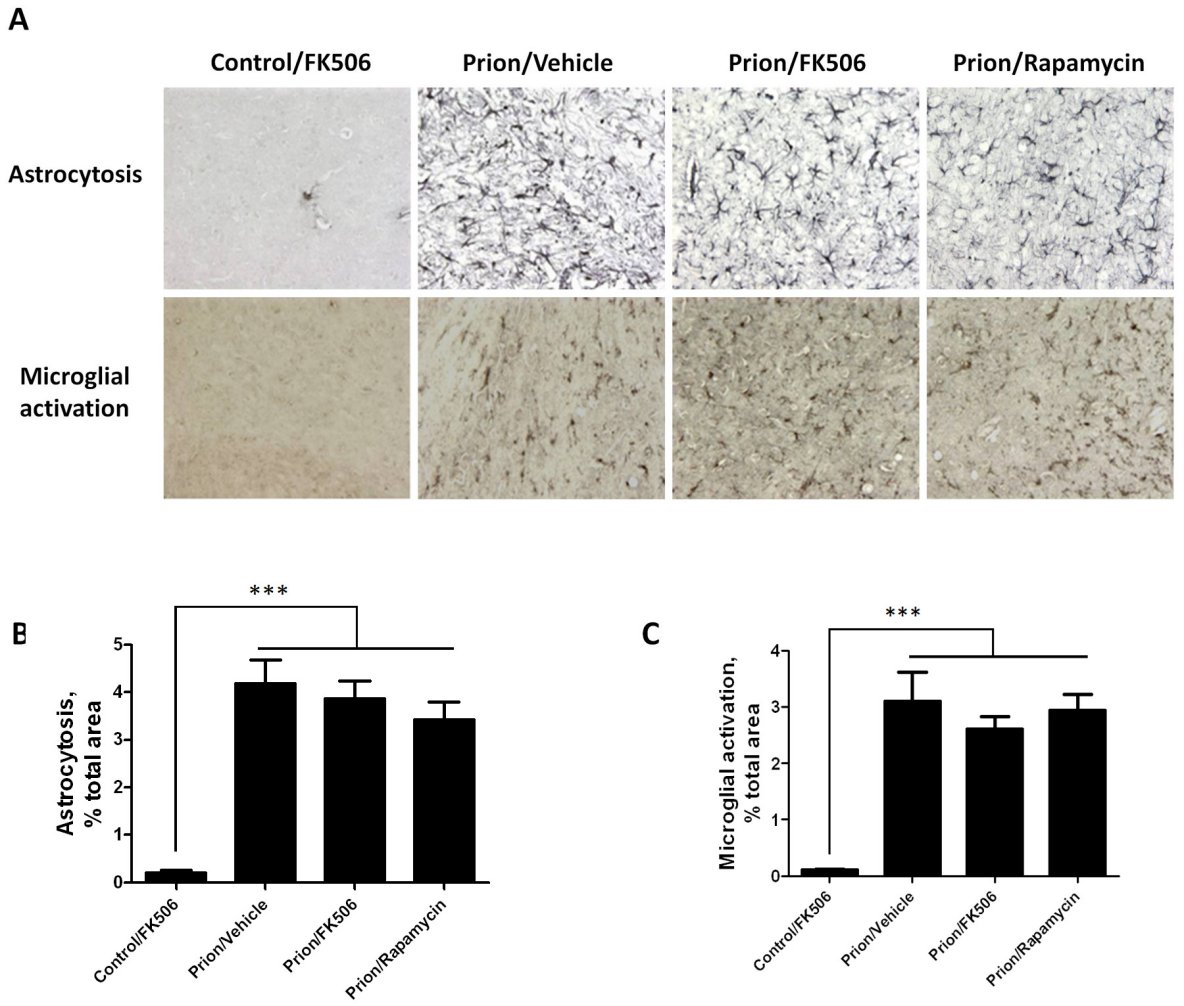
Treatment with FK506 does not alter the extent of astrocytosis or microglial activation. The level of astrocytosis and microglial activation in various brain areas of mice infected or non-infected with prions and subjected to diverse treatments was evaluated by histological analysis. **A**, representative pictures of the thalamus region after staining with GFAP (top) and AIB-1(bottom) to stain reactive astrocytes and activated microglia, respectively. We focused on thalamus, because this is one of the brain areas most severely affected in RML-affected animals. To quantitatively assess the extent of astrocytosis (B) and microglial activation (C) six sections of thalamus were analyzed using the image analysis program Image J from NIH. The values correspond to the stained area expressed as a percentage of the total tissue area. Each value corresponds to the average and standard error (n = 3 for rapamycin group, and n = 4 for all other groups). The data was statistically analyzed by One-way ANOVA followed by the Tukey's Multiple comparison post-test. *** P<0.001.

To further assess the effect of FK506 treatment on prion induced neurodegeneration, fixed brains from 4 animals per group were stained and analyzed. The histopathological alterations observed in prion affected animals, include spongiform degeneration, astroglyosis, neuronal death and synaptic dysfunction. Analysis of the extent of vacuolation revealed no significant differences between treated and un-treated animals (Figure S1). Although, spongiosis is the most characteristic brain alterations observed in TSEs, its role in brain dysfunction and clinical disease is mostly unclear [24], [25]. Brain inflammatory changes in the form of reactive astrocytes and activated microglia are also a typical alteration associated to prion diseases [26]. Since CaN has been implicated in immunological and inflammatory pathways [27], we wanted to analyze in detail the effect of FK506 treatment on the extent of astrocytosis and microglial alteration. Astrogliosis was studied by staining the tissue with the anti-GFAP (Glial fibrillary acidic protein) antibody and reactive microglia with the anti-AIF1 (allograph inflammatory factor 1) antibody (Fig. 5A). Quantification of the area of the thalamus containing reactive astrocytes (Fig. 5B) and activated microglia (Fig. 5B) revealed a pronounced difference between samples coming from non-infected and prion infected animals. However, no statistically significant differences on the extent of brain inflammation were observed among prion infected animals treated or untreated with FK506 or rapamycin. These results suggest that the therapeutic effect of FK506 is not mediated by a neuroimmune pathway.

**Figure 6:**
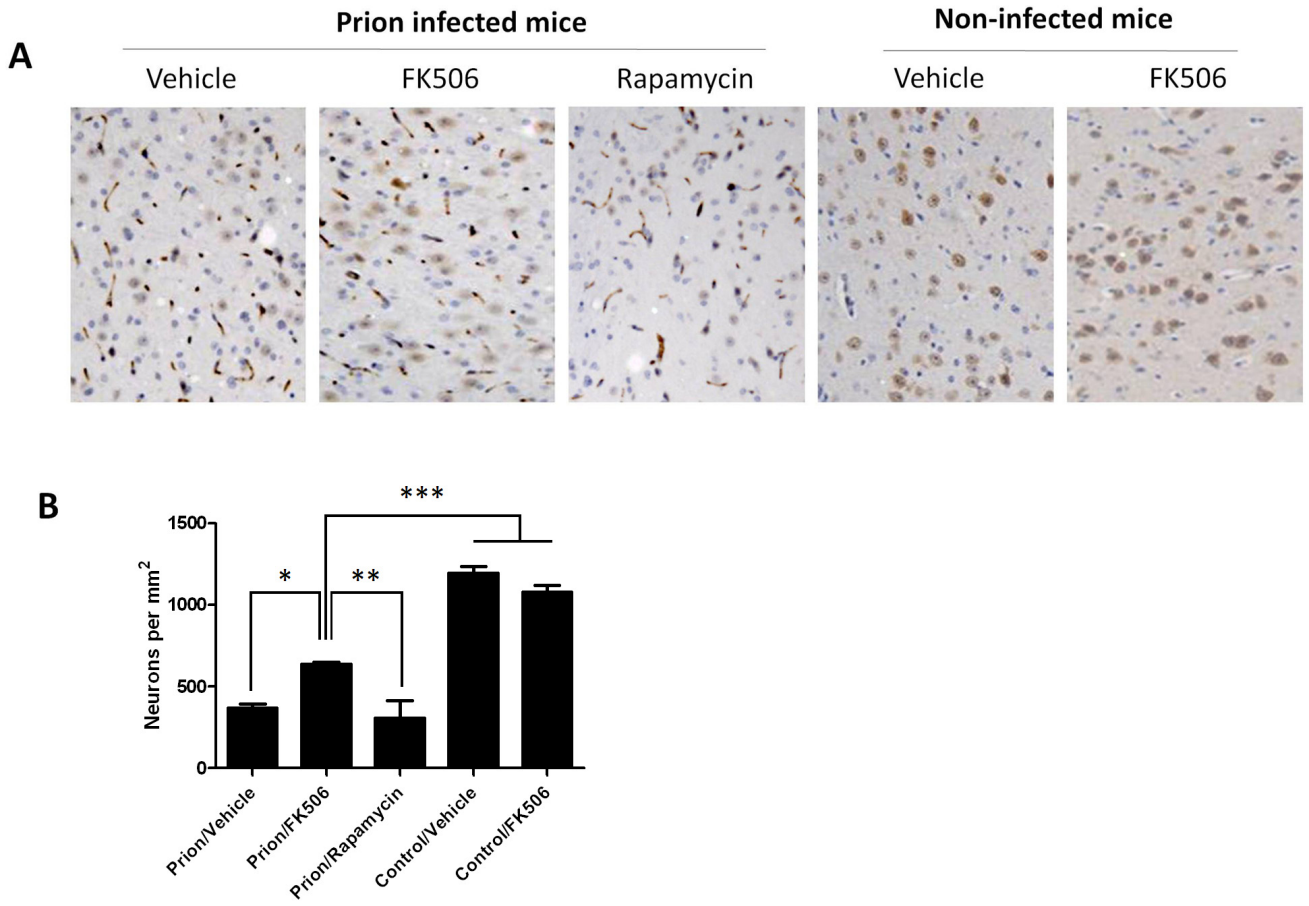
Treatment with FK506 decreases prion-induced neuronal loss. The number of neurons in various brain areas of mice infected or non-infected with prions and subjected to diverse treatments was evaluated by histological analysis using the Neu-N antibody, which specifically recognizes neuronal cells. **A**, representative pictures of the thalamus region after staining with Neu-N (brown) and Hoesch (blue) to stain nucleus. We focused on thalamus, because this is one of the brain areas most severely affected in RML-affected animals. **B**, to quantitatively assess the number of neurons in different groups we randomly selected two different regions of thalamus in each animal and two investigators blinded to the treatment counted the number of neurons using the image pro software. In prion infected animals we observed some abnormal immunoreactivity in the form of elongated structures. We believe this staining represents degenerated nerve cells process or cellular debris. We did not consider these immunoreactivities in the neuronal counting. Each value corresponds to the average and standard error (n = 3 for rapamycin group, and n = 4 for all other groups). The data was statistically analyzed by One-way ANOVA followed by the Tukey's Multiple comparison post-test. * P<0.05; ** P<0.01; *** P<0.001.

**Figure 7:**
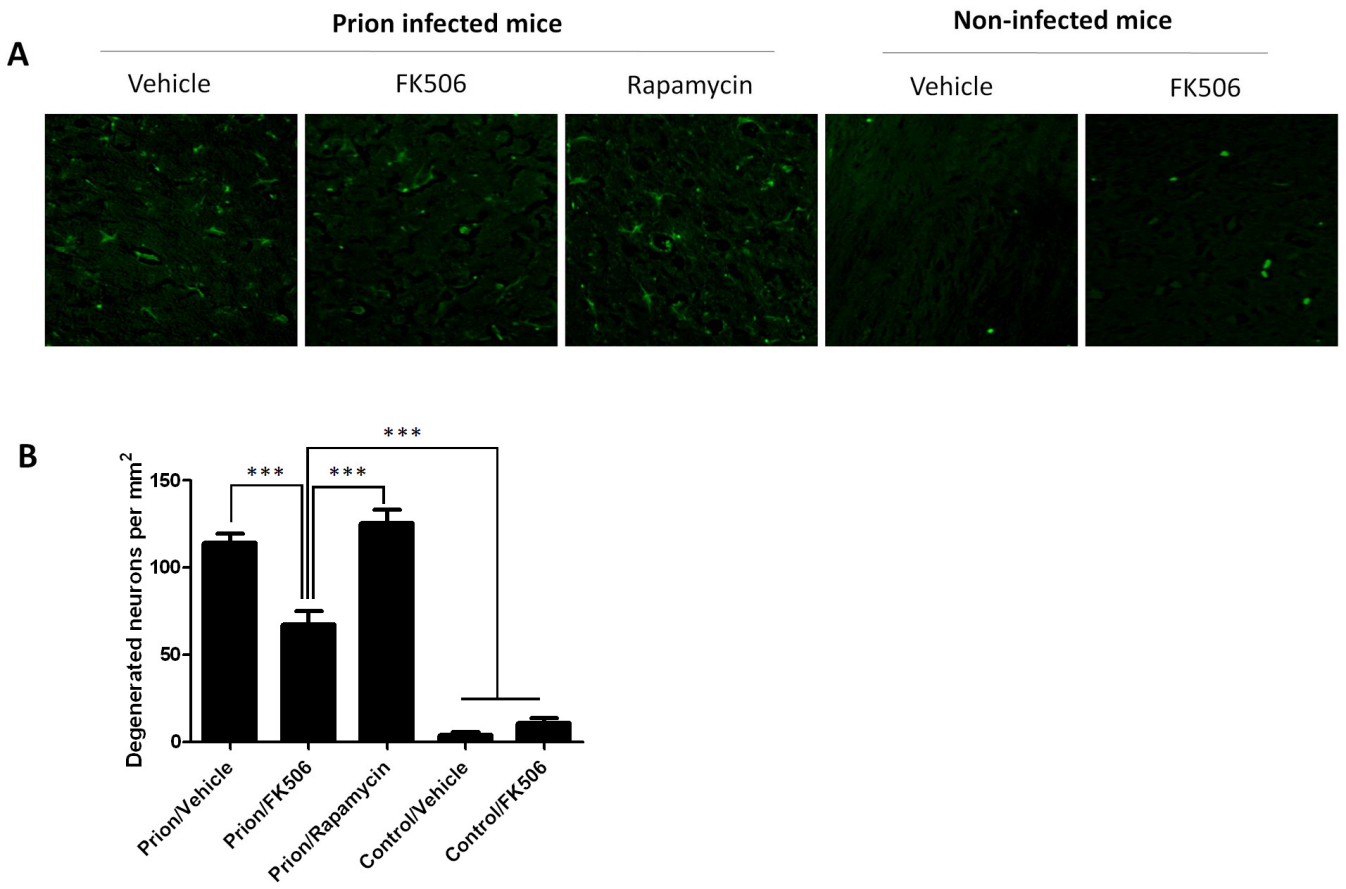
CaN inhibitor reduces neurodegeneration in prion affected animals. Nerve cell degeneration was studied by Fluoro-Jade staining in thalamus sections and visualized by fluorescence microscopy. Panel **A** shows representative pictures from several animals (3 for rapamycin, 4 for the other groups) and panel **B** shows the number of degenerating neurons in each group. The data was statistically analyzed by One-way ANOVA followed by the Tukey's Multiple comparison post-test. *** P<0.001.

To study the number of neurons present in the brain, we stained tissue slides with NeuN, a specific and well-established neuronal marker [28]. The data shows a substantially higher quantity of neurons in the thalamus of FK506 treated-mice, compared with prion affected animals treated with vehicle or rapamycin (Fig. 6). Indeed, mice treated with the CaN inhibitor have almost twice the number of neurons as their untreated-mates. However, still the treated animals have around 50% less neurons than normal animals not affected by prion diseases (Fig. 6), indicating that the treatment only stop in part the neurodegeneration process. In order to further study the influence of the treatment on neuronal damage, we stained the tissue with Fluoro-Jade, a well-established method to detect degenerating neurons [29]. As shown in Figure 7, the brain of mice treated with FK506 has a substantially lower level of Fluoro-Jade stained cells as compared with animals treated with vehicle or rapamycin. Again, the effect of the treatment was not complete, since FK506 treated mice still have significantly more degenerating neurons than control non-infected animals (Fig. 7).

## Discussion

TSEs are dreadful diseases that produce a 100% fatality rate and a progressive and rapid deterioration which leads to complete disability. Currently, there is no therapy available against prion disorders [1]. The self-propagating protein misfolding process that features prion diseases amplifies the toxic and infectious prion in a logarithmic scale making it difficult for development of an efficient therapy at the symptomatic phase. Since there still not available a test to diagnose prion disease at the pre-symptomatic stage [30], the top priority is to develop strategies that could be beneficial after the patients show the first clinical signs of the disease.

Considering that a central event in TSEs is the conversion of PrP^C^ into PrP^Sc^, a widely pursued therapeutic strategy has been to disrupt PrP^Sc^ formation. This approach has been extensively explored by many groups and some compounds have been identified with activity *in vitro* and *in vivo*. The list of compounds studied include: Polyanionic molecules, dextran sulphate, pentosan polysulphate, heparin sulphate mimetics, Phosphorothioate oligonucleotides, congo red analogs, suramin, curcumin, quinacrine, dendritic polyamines, tetracycline, amphotericin B, beta-sheet breaker peptides, anti-PrP antibodies, etc (for reviews, see [8], [9], [31]). However, these compounds produce a benefit mostly when they are administered during the pre-symptomatic stage of the disease, long before the appearance of clinical symptoms. Despite of the lack of positive results in animal models at the clinical phase of the disease, the unmet need for a medicine to treat patients resulted in human clinical trials with at least 3 of these drugs: quinacrine, amphotericin B and pentosan polysulphate [9]. Quinacrine, chlorpromazine, and some of their tricyclic derivatives were described as efficient inhibitors of PrP^Sc^ formation in murine neuroblastoma cells chronically infected with scrapie [32], [33]. However, subsequent animal experiments failed to demonstrate efficacy in the treatment of TSEs [34], even after intraventricular infusion [35]. Because quinacrine and chlorpromazine have been used in human medicine as anti-malarial and anti-psychotic drugs, respectively, they were tested in small clinical trials. No therapeutic effect was seen following quinacrine treatment in two independent trials, although some transient improvement occasionally occurred [36], [37]. Amphothericin B and some of its analogues inhibited prion replication in infected cell cultures [38] and delayed the appearance of spongiosis, astrogliosis, and PrP^Sc^ accumulation in the brain of scrapie-infected hamsters [39], [40]. However, an attempt to treat a CJD patient with amphothericin B was unsuccessful [41]. In view of its high systemic toxicity, these results decrease hopes that amphothericin B will prove useful in prion disease therapy. Several *in vitro* and *in vivo* studies have suggested pentosan polysulphate may be useful in prion diseases [35], [42]–[44]. Pentosan polysulphate is marketed in some countries as a treatment for interstitial cystitis and as anticoagulant, although its side effects include hemorrhage and hypersensitivity reactions. The main problem for using this drug for TSEs is that it does not cross the blood-brain barrier, so it has to be administered either early (during the peripheral prion replication phase) or directly into the brain. Several small observational trials by intra-cerebroventricular infusion of pentosan polysulphate have been conducted, some of them showing promising results [45]–[48]. However, the fact that the drug has to be administered directly into the brain makes its routine use very complicated.

Our approach provides a novel molecular target down-stream of the prion misfolding process and aims to prevent the signaling pathways leading to synaptic alterations and neuronal death. Our data suggests that the pathway by which PrP^Sc^ induces neurodegeneration involves ER-stress, alterations in calcium homeostasis and hyperactivation of CaN, a key brain phosphatase that controls important signaling events modulating neuronal fate and functioning [49]. These findings indicate that down regulation of CaN activity may be a promising target for prion disease therapy. Fortunately, there are known CaN inhibitors extensively studied, such as FK506 and Cyclosporin [21]. FK506 is a FDA approved drug that is used to prevent transplant regression [20]. The drug is sold under the name of Tacrolimus or Prograf. FK506 is produced by Streptomyces tsukubaensis [50]. By inhibiting CaN, the drug suppresses both humoral and cellular immune responses. As an FDA approved drug, the pharmacological properties of the compound are very well-known.

Our results show that administration of FK506 during the symptomatic phase of the disease produced a significant delay of the disease progression manifested as an improvement on behavioral abnormalities and increase survival time compared to controls treated with vehicle. The effect is not dependent on the immunosupresant activity of FK506, since rapamycin (a drug with similar immunosupressive effect, but not acting through CaN) [51] did not produce any change on prion disease. Moreover, administration of FK506 did not alter the pattern of brain inflammation, suggesting that the beneficial effect of this compound is not mediated by neuroimmune pathways. Treatment with FK506 led to a significant increase of pCREB and pBAD levels in the brain, which paralleled the decrease of CaN activity. Importantly, we observed lower degree of neurodegeneration in animals treated with the drug, which was revealed by a higher number of neurons and a lower quantity of degenerating nerve cells. These changes were not dependent on PrP^Sc^ formation, since the protein accumulated in the brain to the same levels as in the untreated mice.

Interestingly, it has been reported that neurodegeneration in other brain diseases associated to protein misfolding also involves ER-stress, changes on calcium homeostasis and CaN activation [52]–[55]. Recent studies have shown an increase in calcineurin signaling during the early clinical symptoms of Alzheimer's disease [56]. Strikingly, administration of FK506 to transgenic mice models of Alzheimer's disease restore memory deficits associated to the accumulation of amyloid-beta oligomers [57], [58] and reduced long-term potentiation deficits produced by aggregated amyloid-beta [59]. These findings indicate that CaN may play a general role in neurodegenerative diseases and could serve as a novel target for therapeutic intervention in these devastating diseases.

## Methods

### Ethics statement

All animal experiments were approved by and conducted in strict accordance with guidelines of the Animal Care and Use Committee of the University of Texas Medical Branch in Galveston and the University of Texas Health Science Center in Houston and complied with the recommendations in the Guide for the Care and Use of Laboratory Animals of the National Institutes of Health.

### FK506 preparation

FK506 was purchased as a powder from LC Laboratories. Purity was >95%. F506 stock solution (0.5 mg/ml) was prepared by dissolving the compound in saline (0.9% NaCl) containing 1.25% PEG40 Castor Oil (Spectrum) and 2% ethanol. FK506 stock solution was stored frozen. Rapamycin (LC Laboratories) was prepared in the same conditions.

### CaN activity assay

The phosphatase activity of CaN was measured using the Calcineurin Cellular Activity Assay kit from Calbiochem as previously described [57]. The brain homogenate were prepared in the assay buffer and the residual phosphate was removed by passing through a desalting column. A final concentration of 1ug/ul of the homogenate was used for the enzyme assay in presence of bovine calmodulin. The reaction mixture was incubated with a final concentration of 150mM RII peptide (substrate) at 37°C for 20 min and reactions were terminated by the addition of 100 µl malachite green reagent. Product formation was measured by recording the absorbance at 635 nm.

### Purification of PrP^C^ and PrP^Sc^

As a source of PrP^C^ we used mouse PrP recombinantly expressed in bacteria, purified and folded into the native structure using a previously described protocol [60]. Briefly, the murine *prnp* gene (coding residues 23–230) was PCR-amplified from mouse blood, inserted in a vector and used to transform BL21-Star *E. coli* cells (Invitrogen). For purification, cell pellets were thawed and resuspended, cells were lysed by adding 0.5 mg/mL Lysozyme and subsequently sonicated. The released inclusion bodies were pelleted by centrifugation and solubilized in buffer containing 10 mM B-Mercaptoethanol and 6M GdnCl. PrP^C^ was purified by using Ni Sepharose 6 Fast Flow resin (GE Healthcare) in batch-mode. Recombinant PrP^C^ was on-column refolded for 6 h and eluted with 500 mM Imidazole. The main peak was collected and quickly filtered to remove aggregates. The protein was confirmed to be monomeric and folded by size exclusion chromatography, Western blotting and Circular Dichroism.

PrP^Sc^ was purified from the brain of RML scrapie sick mice, following a previously described protocol [11]. Briefly, brains were homogenized in PBS and after a low speed centrifugation, samples were mixed with 1 volume of 20% sarkosyl. Samples were centrifuged at 100,000×g for 3 hr at 4°C. Supernatant was discarded; pellets were resuspended, layered over a 20% sucrose cushion and centrifuged for 3h at 4°C. The supernatant was discarded and the pellet resuspended, sonicated and incubated with PK (100 µg/ml) for 2 h at 37°C and shaking. The digested sample was layered over PBS containing 20% Sarkosyl, 0.1% SB 3–14 and 10% NaCl and centrifuged for 1h 30 min at 100.000×g. The final pellet was resuspended in 100 µl of PBS and sonicated. The sample was stored at −80°C. Purity was >90% as analyzed by silver staining and amino acid composition analysis.

### Cell toxicity studies

N2A cells were cultured in DMEM supplemented with 10% fetal calf serum and antibiotics (10'000U/ml Penicillin, 10µg/ml streptomycin), at 37°C and 5% CO2. For cell viability analysis, cells were grown in collagen IV coated 96-well plates for 24h in cell culture medium containing 1% serum before addition of the agonist. Cell viability was quantified using 3-(4,5-dimethylthazol-2-yl)-5-3-carboxymethoxy-phenyl)-2-(4-sulfophenyl)-2H-tetrazolium (MTS) and phenazine methosulfate (PMS) according to the recommendations of the supplier (Promega). Cell death was determined measuring the amount of LDH released to the culture media using the Cytotox 96 LDH kit, following the manufacturer specifications (Promega).

### Determination of cytoplasmatic calcium

The changes in intracellular calcium levels were measured 20 minutes after adding the reagents using the Fluo-4 Direct Calcium Assay kit (Invitrogen) in 96 well plate according to manufacturer's protocol.

### Animal model of prion diseases

The infectious model of prion disease in mice is a very good model for TSEs, since it reproduces many of the clinical, neuropathological and biochemical aspects of the disease in humans and other mammals [61]. Wild type C57Bl6 mice were injected intraperitoneally (i.p.) with 100µl of brain homogenate containing infectious prions (RML strain). Approximately 210 days after inoculation, animals begin to show signs of scrapie. The disease onset was monitored weekly by visual inspection, using the following scale [62]: 1, normal animal; 2, roughcoat on limbs; 3, extensive roughcoat, hunckback, and visible motor abnormalities; 4, urogenital lesions; and 5, terminal stage of the disease in which the animal presented with cachexia and lies in the cage with little movement. Usually animals die few days after reaching stage 5. The period between the appearance of the first disease symptoms and death range between 20–40 days without treatment. Some of the animals were left to die to determine survival time and others were sacrificed at the indicated time by exposure to CO_2_ inhalation to assess brain alterations. After animal sacrifice half of the brain was fixed for histological analysis and the other half was kept frozen for biochemical assays.

### Animal treatment

Treatment with FK506, rapamycin or vehicle was started when animals exhibit the first signs of prion disease (stage 1 in our scale). Animals were injected intra-peritoneally with 0.12 mg of the drug (5 mg/Kg) dissolved in 100 µl of the vehicle solution mentioned above. Administration was done daily until animals die or were sacrificed for experiments.

### Animal behavioral studies

To evaluate if treatment with FK506 alters clinical signs we performed Open field and rotarod tests. The Open field test monitor exploratory behavior and locomotor activity. Animals were placed in a corner of the field box and all activity during various 20s intervals was recorded by a video camera mounted above the open field and scored in real-time. We measured and analyzed total distance, average speed, time spent in various parts of the field, rearing activity and inactive time. Testing was carried out in a temperature, noise and light controlled room. Rota-rod test is used to measure the motor activity and coordination. Animals were placed on the rotating rod with an accelerated speed (initial velocity 5 RPM; acceleration of 3) and the total time spent on the rod was measured. The animal falls from a high of about 6 inches into a plastic platform that automatically counts the time spent in the rod.

### PrP^Sc^ detection assay

The presence and quantity of PrP^Sc^ in brain homogenates of sick animals was measured by a standard assay consisting of the ability of the misfolded protein to resist proteolytic degradation. Samples were incubated in the presence of proteinase K (50 µg/ml) during 60 min with shaking at 37°C. The digestion was stopped by adding electrophoresis sample buffer and protease-resistant PrP was detected by western blotting, as previously described [63]. Briefly, proteins were fractionated by sodium dodecyl sulphate-polyacrylamide gel electrophoresis (SDS-PAGE), electroblotted onto nitrocellulose membrane and probed with 6D11 antibody at a 1∶5,000 dilution. The immunoreactive bands were visualized by enhanced chemoluminesence assay (Amersham) and densitometric analysis done by using a UVP image analysis system.

### Detection of pCREB and pBAD

Frozen brain samples were homogenized in RIPA buffer containing a cocktail of protease inhibitors and were sonicated for 15 s, and then centrifuged at 20,000 g for 5 min. The supernatants were collected and protein concentration measured using BCA assay (Pierce). 50µg of protein extracts were subjected to SDS-PAGE, and western blotting. The membrane was immunoblotted with pBAD, pCREB (Cell signaling; 1∶1000) antibodies and the target proteins were subsequently detected using horseradish-peroxidase conjugated anti-IgG secondary antibodies (Amersham Biosciences; 1∶2000). Then the membrane was stripped and reblotted with BAD, CREB (cell signaling; 1∶1000) to determine the total protein level. In all cases the membrane was reprobed with β-actin (Cell signaling;1∶5000) to ensure equal protein loading. As before gels were densitometrically analyzed by the UVp image analysis system.

### Postmortem neuropathological analysis

Histological studies were done to assess the effect of the treatment on brain damage. For this purpose half of the brain was fixed in 10% formaldehyde solution, embedded in paraffin and cut in sections using a microtome. Serial sections (8 µm thick) from each block were stained to assess PrP deposition, spongiform degeneration, brain inflammation, neuronal degeneration and neuronal loss. The following studies were done: a) *Brain Vacuolation*. One of the neuropathological hallmarks of prion diseases is the presence of spongiform degeneration in the brain. Vacuolation was assayed by staining of the tissue with Hematoxilin and eosilin. Then, the number of vacuoles was counted in cerebellum, hippocampus, inferior culliculum, occipital cortex, frontal cortex and thalamus of each animal, as described [62]. b) *Brain inflammation*. Astrocytosis was assayed by immunohistochemistry utilizing antibodies against Glial Fibrillary Acidic Protein (GFAP) expressed in abundance in activated astrocytes, following a previously described protocol [62]. Staining for activated microglia was done with the AIF-1 antibody. AIF-1 is a 17 kDa interferon-gamma inducible calcium binding protein, associated with chronic inflammatory processes, which has been previously used to assess microglial activation in CJD patients [64]. Digital images were collected on a Leica Microscope fitted with an apotome for optimal sectioning. To calculate the extent of astrocytosis and microglial activation, six 20× sections of Cortex, Thalamus, Hippocampus and Cerebellum were collected per animal and the stained area compared to the total tissue area was determined using the image analysis program Image J from NIH. c) *Neurodegeneration*. To evaluate neuronal degeneration and death we used Fluoro-JadeB staining to detect degenerative cells and NeuN, a specific marker for neurons. The Fluoro-Jade B staining was done following the protocol described by Liu et al [65]. Briefly, 8 µm sections were cut from paraffin embedded brains and spread on microscope slides and allowed to air dry followed by mounting on microscope slides and placed in 70% ethanol. The sections were washed and oxidized by soaking in a solution of 0.06% KMnO4 for 15min. After washing, they were stained with 0.001% Fluoro-JadeB (MiliPore) in 0.1%acetic acid for 20min. Slides were washed again and dried overnight at room temperature. Digital images were collected on a Leica Microscope fitted with an apotome for optimal sectioning. Six 20× sections of Cortex, Thalamus, Hipocampus and Cerebellum were collected per animal. Fluoro-Jade B positive cells were counted from each field. The number of total neurons was counted after staining with the monoclonal anti-NeuN antibody (Chemicon) at 1/1000 dilution. NeuN is a specific neuronal marker for a DNA-binding protein present in the nucleus of postmitotic neurons [28]. Brain sections were mounted onto gelatin-coated coverslips and allowed to air dry. Air dried sections were blocked and permeabilized in 0.1MPB with 0.3% TX-100 (Sigma) and 10% goat serum (PBTGS) for 1 h. Following permeabilization, the mouse monoclonal anti-NeuN antibody (Chemicon International) was applied at a 1∶200 dilution and incubated overnight at room temperature. After washing, the secondary antibody and Hoechst (Molecular Probes) were applied for 1 h at room temperature followed by 3 consecutive washes. Slides were visualized under the microscope by two different researchers blinded to the treatment who counted the number of neurons in different brain areas.

### Statistical analysis

For the *in vitro* studies of the effect of PrP^Sc^ on calcium, CaN activity and cell death, the data was analyzed by one-way ANOVA, followed by the Tukey's Multiple Comparison post-test to estimate the significance of the differences. The *in vivo* survival study was assessed by the Log-rank (Mantel-cox) test. The effect of treatment on the rotarod performance was evaluated by two-ways ANOVA using time and treatment as the variables. The behavioral study by open field test and the differences on CaN activity in animals at different times post-inoculation were evaluated by unpaired t-test (two-tailed). Finally, the effect of treatment on the CaN activity, CREB phosphorylation, BAD phosphorylation, astroglyosis, microglial activation, number of neurons and degenerating cells was analyzed by one-way ANOVA, followed by the Tukey's Multiple Comparison post-test to estimate the significance of the differences. All statistical analysis was done with the GraphPad Prism, version 5.0 software.

## Supporting Information

Figure S1 Spongiform degeneration in the brain of prion-infected mice treated with FK506. The vacuolation extent and distribution was evaluated after hematoxilin-eosin staining of fixed section. The figure shows representative pictures of 3 brain areas (cerebellum, medulla, hippocampus) of various animals analyzed in the different groups.
(0.35 MB PDF)
