# Supplementary material for: Calcineurin Inhibition at the Clinical Phase of Prion Disease Reduces Neurodegeneration, Improves Behavioral Alterations and Increases Animal Survival
Source: PLoS Pathog. 2010 Oct 7;6(10):e1001138. doi: 10.1371/journal.ppat.1001138 (PMC2951383; doi:10.1371/journal.ppat.1001138)

Supplementary Information

Supplementary Figure 1. Spongiform degeneration in the brain of prion-infected mice treated with FK506. The vacuolation extent and distribution was evaluated after hematoxilin-eosin staining of fixed section. The figure shows representative pictures of 3 brain areas (cerebellum, medulla, hippocampus) of various animals analyzed in the different groups.

Supplementary Figure 1

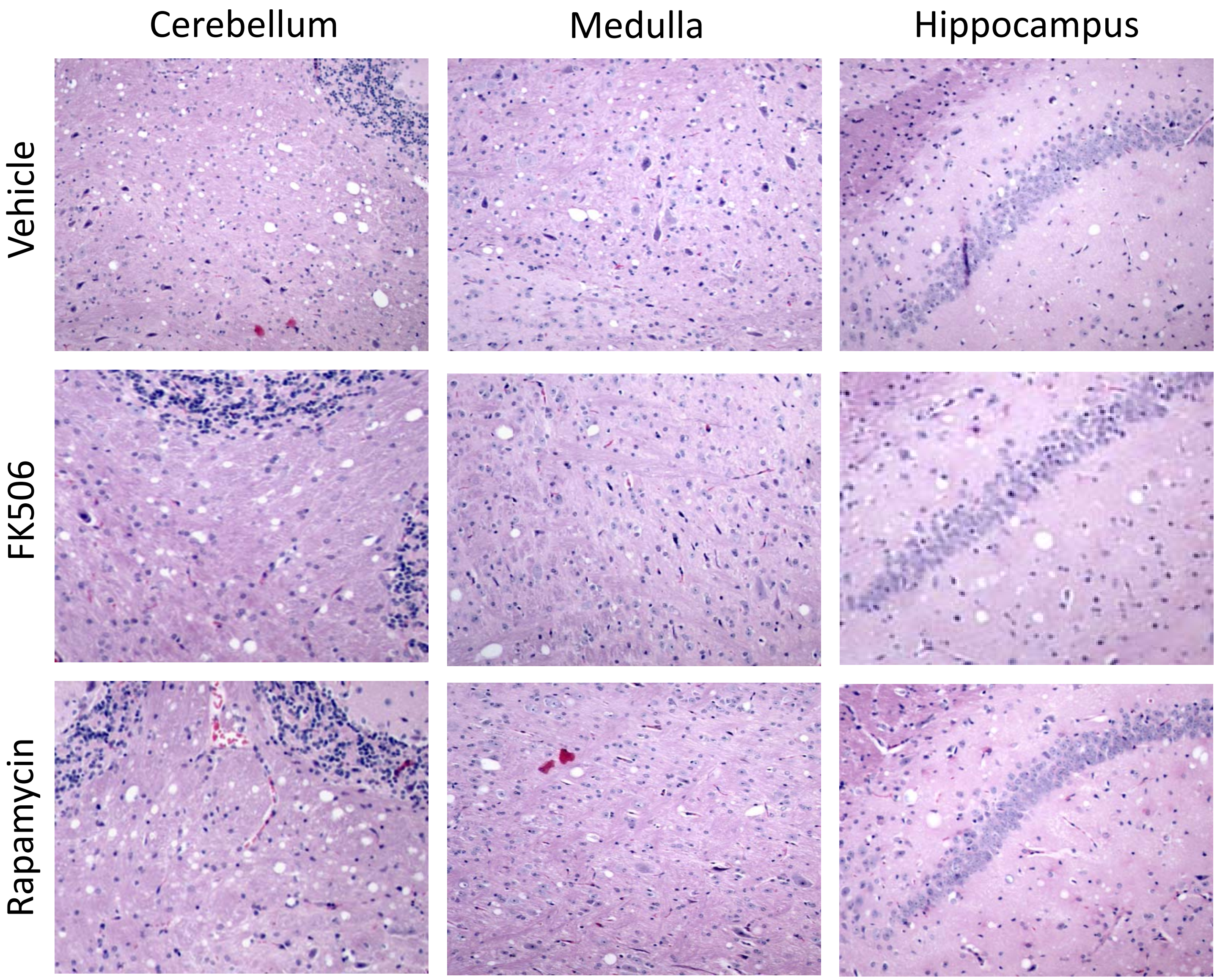

Supplement: Figure S1 — Spongiform degeneration in the brain of prion-infected mice treated with FK506. The vacuolation extent and distribution was evaluated after hematoxilin-eosin staining of fixed section. The figure shows representative pictures of 3 brain areas (cerebellum, medulla, hippocampus) of various animals analyzed in the different groups. (0.35 MB PDF) [file ppat.1001138.s001.pdf]
